# Automatic Skeleton Segmentation in CT Images Based on U-Net

**DOI:** 10.1007/s10278-024-01127-5

**Published:** 2024-04-30

**Authors:** Eva Milara, Adolfo Gómez-Grande, Pilar Sarandeses, Alexander P. Seiffert, Enrique J. Gómez, Patricia Sánchez-González

**Affiliations:** 1 Biomedical Engineering and Telemedicine Centre, Center for Biomedical Technology, ETSI Telecomunicación, Universidad Politécnica de Madrid, 28040 Madrid, Spain; 2 Department of Nuclear Medicine, Hospital Universitario, 12 de Octubre, 28041 Madrid, Spain; 3 Facultad de Medicina, Universidad Complutense de Madrid, 28040 Madrid, Spain; 4 Centro de Investigación Biomédica en Red de Bioingeniería, Biomateriales y Nanomedicina, Instituto de Salud Carlos III, 28029 Madrid, Spain

**Keywords:** Bone automatic segmentation, Neural networks, U-Net architecture, Anatomical images

## Abstract

Bone metastasis, emerging oncological therapies, and osteoporosis represent some of the distinct clinical contexts which can result in morphological alterations in bone structure. The visual assessment of these changes through anatomical images is considered suboptimal, emphasizing the importance of precise skeletal segmentation as a valuable aid for its evaluation. In the present study, a neural network model for automatic skeleton segmentation from bidimensional computerized tomography (CT) slices is proposed. A total of 77 CT images and their semimanual skeleton segmentation from two acquisition protocols (whole-body and femur-to-head) are used to form a training group and a testing group. Preprocessing of the images includes four main steps: stretcher removal, thresholding, image clipping, and normalization (with two different techniques: interpatient and intrapatient). Subsequently, five different sets are created and arranged in a randomized order for the training phase. A neural network model based on U-Net architecture is implemented with different values of the number of channels in each feature map and number of epochs. The model with the best performance obtains a Jaccard index (IoU) of 0.959 and a Dice index of 0.979. The resultant model demonstrates the potential of deep learning applied in medical images and proving its utility in bone segmentation.

**Supplementary Information:**

The online version contains supplementary material available at 10.1007/s10278-024-01127-5.

## Introduction

Segmentation of the skeleton from computerized tomography (CT) images is very useful in clinical practice in multiple clinical contexts. Being the bone the most common and prevalent metastasis site, mainly in breast and prostate cancer, bone segmentation could improve its evaluation by morphological change detection [1, 2 ], even more so when the visual assessment of morphological images for the evaluation of the response of bone metastases is considered inadequate according to the Response Evaluation Criteria In Solid Tumors 1.1 (RECIST1.1) [3]. In addition, the assessment of the response to new oncological therapies, such as radioligand therapy, or the evaluation of osteoporosis can benefit from the application of bone segmentation in fracture or weakness examination [4–6].

Bone segmentation from CT images is usually obtained through manual processes. These are not only time-consuming but also subject to interoperator variability. However, in recent years, there are arising new automatic skeletal segmentation algorithms based on CT images. Pérez-Carrasco et al. [7] developed an algorithm based on energy minimization for the segmentation of bone and muscle structures. For this purpose, a dataset of 27 CT volumes is manually segmented for training and testing the models. For preprocessing the images, skin delimitation by a region-growing algorithm from random seeds is performed. Then, bone and muscles are segmented by means of an energy minimization algorithm. The segmentation algorithms are divided in three main steps: thresholding and scaling the CT volumes to differentiate bone and muscles, computation of histogram distance images, and continuous max-flow segmentation algorithm. Finally, a bone segmentation with a Jaccard index or Intersection over Union (IoU) of 0.8 and a Dice index of 0.88 is obtained.

Not only conventional image processing algorithms are applied for skeleton segmentation but also deep learning models, which have been widely extended. Klein et al. [8] implement, with a dataset of 18 CT volumes, a convolutional neural network with an architecture based on U-Net [9] for whole-body skeleton segmentation. With 64 channels for the first feature map, the resulting model obtains a segmentation of the skeleton from the 2D slices with a Jaccard and Dice index of 0.91 and 0.95, respectively. In line with this study, Noguchi et al. [10] implement a very similar model with the difference of using a dataset of 32 CT volumes and 16 channels for the first feature map. In addition, they use a robust data augmentation methodology including the conventional methods, such as rotation, zooming, flip, or shear transformation, and the mix-up method, a technique which generates new samples from the combination of existing images and their labels. Despite the simpler model, the performance outperforms the previous one, reaching values of 0.968 and 0.983 for the same indexes. Although there are multiple studies proposing skeletal segmentation methodologies, they have not yet been extended to clinical practice.

The main objective of the present study is obtaining an automatic skeleton segmentation from 2D CT slices using the U-Net architecture. For this purpose, models are generated by varying: the preprocessing of the initial image, the input order of the training images, and the parameters that define the model. Once the performance of all the models has been evaluated, the one that achieves the best segmentation is selected.

## Material and Methods

### Subjects

The study cohort was formed by 77 CT images from two different protocols: 45 cases of the multiple myeloma diagnostic protocol (whole-body) and 32 cases of the follicular lymphoma diagnostic protocol (femur-to-head). Siemens Biograph TruePoint 6 PET/CT (Siemens Healthineers, Erlangen, Germany) was used to obtain low-dose CT scans as a part of [^18^F]FDG PET/CT studies at the Nuclear Medicine Department of the Hospital Universitario 12 de Octubre in Madrid. These images were acquired based on the European Association of Nuclear Medicine (EANM) procedure guidelines [11]. CT images were obtained using helical CTs (120–140 kVp, 25–170 mAs) with a resolution of 512 × 512 with a voxel size of 0.9766 × 0.9766 × 2.5 mm^3^. The number of slices of these CT scans varies from 254 to 724.

The ground truth, i.e., the mask skeleton and the output data for the model, was obtained using the tool described in Milara et al. [12]. First, an automatic segmentation of the skeleton is performed. Then, the spinal canal is removed. Finally, a manual edition of the segmented mask is performed using the interface available in the same tool.

### CT Preprocessing

Before using the CT images as model input, a preprocessing consisting of four steps is performed in MATLAB 2021a (The MathWorks Inc., Natick, MA, USA): (1) *stretcher removal*, to prevent the model from confusing the stretcher with the bone due to the similarity in high-intensity levels in the CT image; (2) *thresholding*, to unify the gray intensity levels corresponding to the cortical bone and bone marrow, as well as to unify the region removed from the stretcher with the image background; (3) *image clipping*, to unify lymphoma diagnostic protocol cases with multiple myeloma diagnostic protocol cases; and (4) *normalization*, to achieve a normalization of all cases to the same gray intensity levels. These preprocessing steps are based on the mistakes observed in skeleton segmentation with a model trained with non-preprocessed CT scans (see Supplementary Material. A).

### Stretcher Removal

**Fig. 1:**
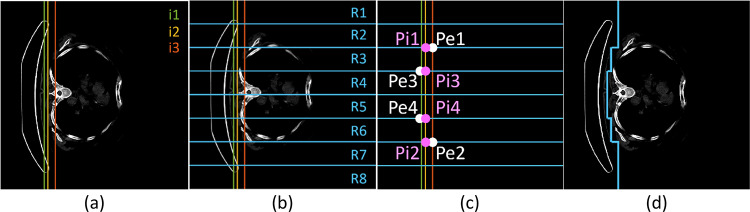
Stretcher removal I: margin location. **a** Location of the vertical lines that separate the stretcher from the body of the patient: in the central zone (i1, green), in the curve that goes from the central zone to the ends of the stretcher (i2, yellow), and at these ends (i3, red). **b** Horizontal division of the slice in eight regions of identical size (R1–R8, blue). **c** Intersection points from Pe1 to Pe4 and Pi1 to Pi4 shown over the correspondent vertical and horizontal lines (CT scan is not included for getting an easier visualization). **d** Margin (blue) separating the stretcher from the skeletal area shown in the CT slice

**Fig. 2:**
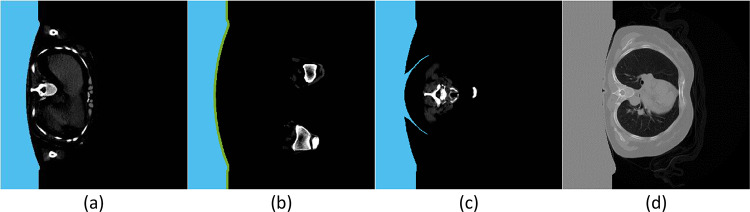
Stretcher removal II: stretcher filling and adaptation. The blue mask represents the stretcher mask. **a** Stretcher-filled mask for a CT slice without adaptation. **b** Stretcher mask for a CT slice with adaptation by means of dilatation. The green mask represents the adaptation with respect to the original stretcher. **c** Stretcher mask for a CT slice with adaptation by means of stretcher accessory searching. **d** Original CT scan slice substituting the values of the stretcher region by 0

Figures 1 and 2 show the step-by-step process of stretcher removal (see the complete process in Supplementary Material. B). First, the CT image is binarized with a threshold of 98 HU. From the binarized image, the point closest to the left image margin is located and defined as the beginning of the stretcher (ancestor of i1). The next margin of the stretcher in the horizontal central zone is located (ancestor of i2). Finally, to include also the extremes of the stretcher, a third index is obtained (ancestor of i3). Figure 1a shows the estimated intermediate vertical lines between these calculated limits to the body (i1, i2, and i3).

Subsequently, the axial slice is divided in its vertical direction into eight equal regions R1–R8 (shown in blue in Fig. 1b). From these horizontal regions, the intersection points between the vertical lines and the horizontal regions are related and divided as follows (Fig. 1c): the extremes of the stretcher (R2 and R7 with i3), the central area of the stretcher (R4 and R5 with i1), and the inclined areas of the stretcher (R3 and R6 with i2). Then, the intersection points are connected as follows: Pe1—Pi1—Pi3—Pe3—Pe4—Pi4—Pi2—Pe2; and finally, the resulting line extends vertically to the lower and upper limits of the image following i3. In this way, the margin separating the stretcher from the skeletal area is obtained (Fig. 1d).

Once the margin separating the stretcher from the skeletal regions is obtained, the space between the stretcher and the left margin is considered a single stretcher mask (Fig. 2a). Finally, this mask is adapted to every slice of the same patient. For this purpose, a dilatation of the mask is performed, reducing the number of interesting-for-segmentation pixels (Fig. 2b), or even detecting some accessories objects of the stretcher (Fig. 2c). Once the stretcher mask is obtained, it is removed from the CT scan defining every pixel present in the stretcher mask as a 0 in the CT scan (Fig. 2d).

### Thresholding

In order to focus the segmentation on the whole bone region, including compact bone and bone marrow, the CT scan was processed using a thresholding method. In this way, all voxels with a value lower than or equal to 0 HU are assigned the value 0 HU, since these HU values in CT correspond to uninteresting elements for analysis such as air (background) and some soft tissues (such as adipose tissue). In addition, to include within the skeleton segmentation both the cortical skeleton and the bone marrow, a value of 300 HU is assigned to all voxels with a value greater than or equal to 300 HU, being the only tissues in the human body with a HU value close to and above 300 on a CT scan, which prevents further tissue mistaking at the upper threshold value.

### Image Clipping

Based on the fact that only 45 CT scans (the multiple myeloma diagnostic protocol studies) include the regions below the femurs and that the segmentation based on the application of Milara et al. [12] starts from the same point, all images have been cropped from the first visible femur slice. In addition, as in the study by Milara et al. [12], the axial slices corresponding to the head are also eliminated because of their lack of interest in the study of bone lesions and due to its susceptibility to movement.

### Normalization Experiments

The intensity normalization of the image volume is the final step of the preprocess. The volume (*V*) is normalized between 0 and 255 by means of the (1), being minGL and maxGL as the minimum and maximum value of the gray level of the input volume, respectively, i.e., 0 and 300 after the thresholding step. Finally, the values are converted into integer values.

1 $${V}_{normalized}= \frac{255 \times (V-minGL)}{(maxGL-minGL)}$$

The importance of using intrapatient or interpatient normalization is assessed. For this purpose, two different experiments are tested differing in the order of the normalization step:

1. *Experiment A*. The normalization step is applied interpatient and intraset, i.e., when the input volume (*V*) is composed of the random axial slices of the random patients.
2. *Experiment B*. The normalization step is applied intrapatient, i.e., when the input volume (*V*) is composed of the axial slices of the same patient. Then, the dataset creation is performed with the normalized volumes of the patients.

### Datasets Creation

**Fig. 3:**
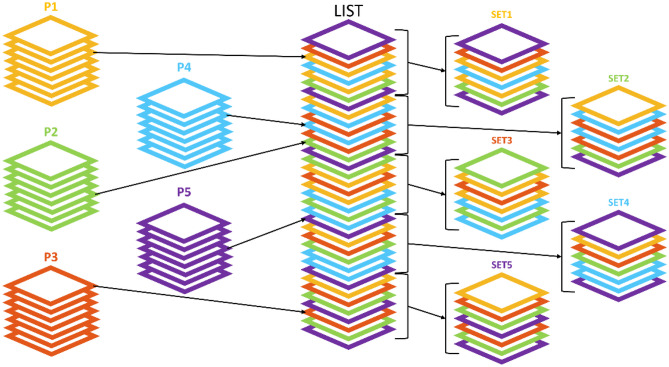
Dataset creation. Example of dataset creation with patient and slice randomization

In order to avoid overfitting the model to images of the same anatomical region, e.g., slices corresponding to the femur region, all training CT image slices, accounting for 65 patients with a total of 21,304 samples, are ordered randomly, both by patient number and by slice number. Figure 3 shows an example of how a dataset is created from the CT scans of five patients instead of the real 65 patients that are used for facilizing its visualization. Firstly, a patient is randomly selected. Secondly, a slice of the CT scan of this patient is randomly selected. The patient and its slice are saved in a shorted list. Then, this process is repeated until there are no slices left in any patient in the training group.

Once the list is completed, it is divided into five different list sets. Every list set is converted into two volumes with the same corresponding slices: one for the CT scans (inputs) and the other for the skeleton mask (outputs). This division avoids creating large files, with 4261 samples in each set, and allows to randomize the input order of the sets too.

For the test group, the set creation is completely different and with the remaining 12 patients of the available study cohort. All 12 CT scans are added to a new list set, test list, slice-by-slice, and patient-by-patient, without randomization, since it does not provide learning to the model. In the same way as for the training group, for the test group, an input volume is created with the 3813 slices of the CT scans, and an output volume with the slices of the skeleton masks following the test list.

Finally, ten different training orders of the model are randomly generated. All sets appear only once in each order. This way, the impact of the order of appearance of the sets on the training of the model is evaluated.

### Model Architecture

**Fig. 4:**
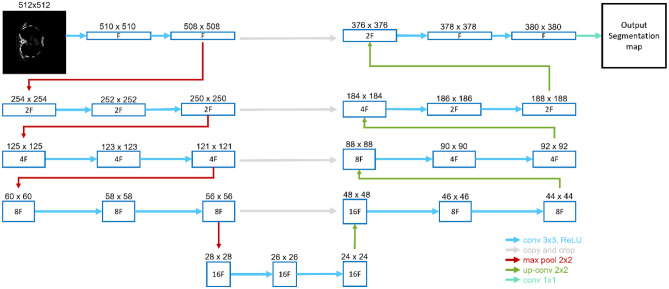
U-Net architecture for input image of 512 × 512 resolution. Each blue box corresponds to a multi-channel feature map. The number of channels is denoted inside the box as follows: F, 2F, 4F, 8F, and 16F. The resolution of each feature map is provided on top of the box. The arrows denote the different operations. The left half of the diagram represents the contractive path and the right half the expansive path

For the skeleton segmentation from the CT image, a 2D model based on the U-Net architecture described in [9] is used. In this case, the number of channels in each feature map is not permanent; it is defined by the parameter *F* as it can be seen in Fig. 4. The model is tested for three different values of this parameter (2, 4, and 8). No higher values are tested due to the GPU limitations (only 6 GB of VRAM). The model is tested with different numbers of epochs (5, 10, and 20) for the training. With the objective of not overfitting the model, a higher number of epochs are not tested. The same model is used for both normalization experiments.

The input image, an axial slice (2D image), is converted in an output segmentation map with the percentage of probability of being part of the binary mask, from 0 to 1, for each pixel. If the probability is higher than or equal to 0.5, this pixel is included in the mask (value = 1). Otherwise, this pixel is interpreted as background (value = 0). In this way, the input image is converted into a binary image conformed of pixels with a value of 1 for the skeleton mask and pixels with a value of 0 for the background (non-skeleton regions, including soft tissues).

Python 3.9.12 software (Python Software Foundation, EEUU) has been used for the implementation, training, and testing of the model. The complete development has been carried out using an NVIDIA GeForce GTX 1660 GPU with 6 GB of VRAM.

### Performance Assessment

For evaluating the quality of the segmentation, the obtained volume is compared to the ground truth. From this comparation, four values are obtained:

- *True positives (TP)*: pixels which appear as part of the skeleton mask in both volumes.
- *True negatives (TN)*: pixels which appear as part of the background in both volumes.
- *False positives (FP)*: pixels which appear as part of the skeleton mask in the predicted volume but are actually background in the ground truth.
- *False negatives (FN)*: pixels which appear as part of the background mask in the predicted volume but are actually a skeleton in the ground truth.

Once the values are obtained, multiple performance metrics are calculated for comparing model performance [13]. First of all, the Jaccard or IoU index for the prediction of the skeleton mask, the background mask, and its mean are calculated by (2), (3), and (4), respectively.

2 $$IoU \left(mask\right)= \frac{TP}{TP+FP+FN}$$

3 $$IoU \left(background\right)= \frac{TN}{TN+FP+FN}$$

4 $$IoU= \frac{IoU \left(mask\right)+IoU (background)}{2}$$

In an attempt to obtain an index that assigns more importance to correctly segmented regions as opposed to erroneous regions, the Dice index is calculated [13]. Using (5), (6), and (7), the Dice index for the skeleton mask segmentation, the background segmentation, and the mean of both are calculated, respectively. Finally, to prove the statistically significant differences between models with different *F* and *E* values, Kruskal–Wallis and post hoc analyses are performed (all results are provided in Supplementary Material. C).

5 $$Dice \left(mask\right)= \frac{2\times TP}{2\times TP+FP+FN}$$

6 $$Dice \left(background\right)= \frac{2\times TN}{2\times TN+FP+FN}$$

7 $$Dice= \frac{Dice \left(mask\right)+Dice (background)}{2}$$

## Results

### Subjects

Once the three first preprocessing steps are applied over the complete database (including stretcher removal, thresholding, and image clipping), the 77 CT scans are divided into two groups: the training group and the test group. The training group consists of 65 images, i.e., 85% of the total. For the remaining 15%, 12 CT scans form the test group. As the created model is trained with the 2D images, the CT scan slices, the training group is formed by 21,304 slices randomly assigned from the 65 patients set aside for the training group. Then, this number of slices is divided into five sets formed by 4261 slices the first four sets and 4260 the last one. On the other hand, the test group consists of 3813 slices from the remaining 12 patients.

### Experiment A Results

**Fig. 5:**
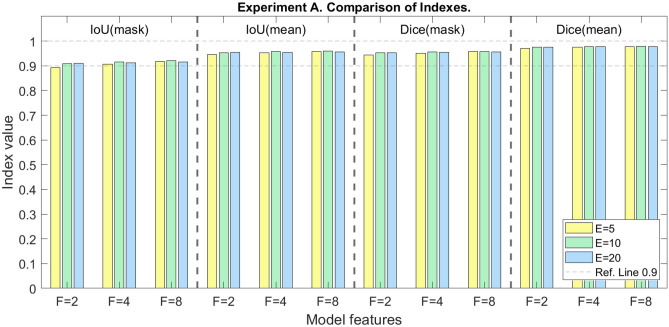
Comparison of Experiment A models. *F*, number of channels in each feature map; *E*, number of epochs in the training process

A different model is created, trained, tested, and assessed for every set of values: *F*, the number of channels in each first feature map; *E*, the number of epochs used in the training process; and training order. Figure 5 shows the results of the model with the highest value of performance metric for each pair of model parameters (*F* and *E*), i.e., the training order with the best results is selected. As it can be seen, all models except the model with *F* = 2 and *E* = 5 obtain a value over 0.9 for all the indexes. Considering the variation of epochs (*E*), all models with *E* = 5 obtain significantly worse performance than models with a higher number of epochs and the same value for the parameter *F* (*p* < 0.001). However, the performance results for models with the same *F* and *E* varying from 10 to 20 do not follow a stable tendency, i.e., a higher value of *E* does not imply a better performance (*p* < 0.001). On the other hand, models with *F* = 2 obtain significantly worse results than the rest of the tested models (*p* < 0.001). However, models with the same *E* but differing between *F* = 4 and *F* = 8 obtain similar results, but with statistically significant differences (*p* < 0.001).

**Fig. 6:**
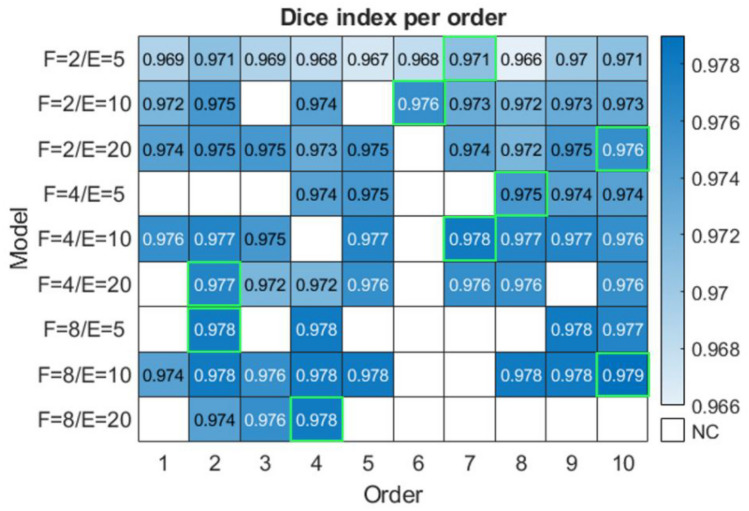
Comparison of training orders for each model, i.e., different pairs of *F* and *E* in Experiment A. NC, non-convergent. In lighting green is shown the best result for each pair of feature models: the best Dice index; a same value of Dice index, the best Dice (mask) index; a same value of Dice (mask) index, the model with higher value of TP

Some sets of values (*F*, *E*, and training order) obtain a model which classifies every pixel as background, i.e., the model fails to converge into a segmentation. Figure 6 shows a heatmap with the Dice index represented for each pair of model parameters (*F* and *E*) vs. training order. Although the simpler the model (lower values of *F* and *E*), the greater the chances of obtaining a convergent model, the results are worse than those of more complex models (higher values of *F* and *E*). Furthermore, it can be observed that there are some training orders (2, 4, and 10) which achieve convergent models for more pairs of model parameters (*F* and *E*), which obtains a convergent model in eight of the feature pairs. However, the best results do not always correspond to these orders. For example, the seventh order obtains better results for two parameter pairs in spite of being non-convergent for four pairs. A total of 62 (69%) convergent models are produced in Experiment A.

### Experiment B Results

**Fig. 7:**
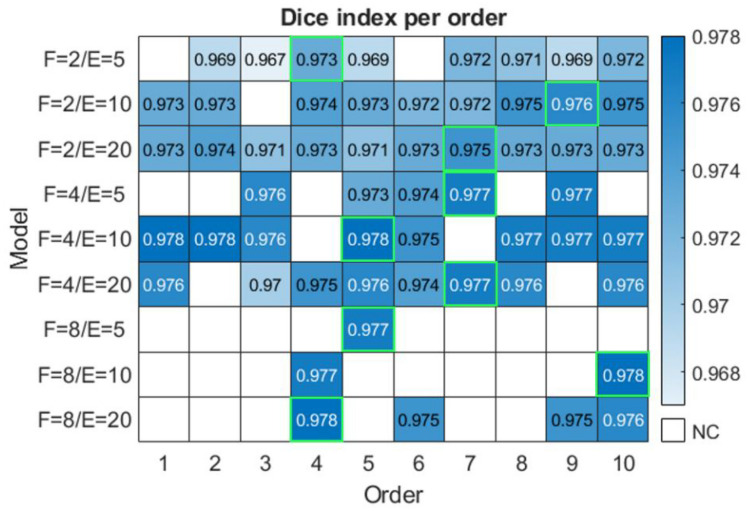
Comparison of training orders for each model, i.e., different pairs of *F* and *E* in Experiment B. NC, non-convergent. In lighting green is shown the best result for each pair of feature models: the best Dice index; a same value of Dice index, the best Dice (mask) index; a same value of Dice (mask) index, the model with higher value of TP

As in Experiment A, a different model is created, trained, tested, and assessed for every set of values: *F*, *E*, and training order. In Fig. 7, the performance of the models obtained for every set of values in Experiment B is shown. Comparing to Fig. 6, it can be observed that the performance obtained in Experiment B is very similar although, in general, slightly lower than those of Experiment A. However, there is some tendency for the best-performing models (the more complex ones due to their higher *F* value) to be slightly higher for Experiment A, while the simpler ones are slightly higher for Experiment B. In addition, it can also be observed that Experiment B achieves a lower number of convergent models than Experiment A with a total of 55 (61%).

### Best Model Selection

**Fig. 8:**
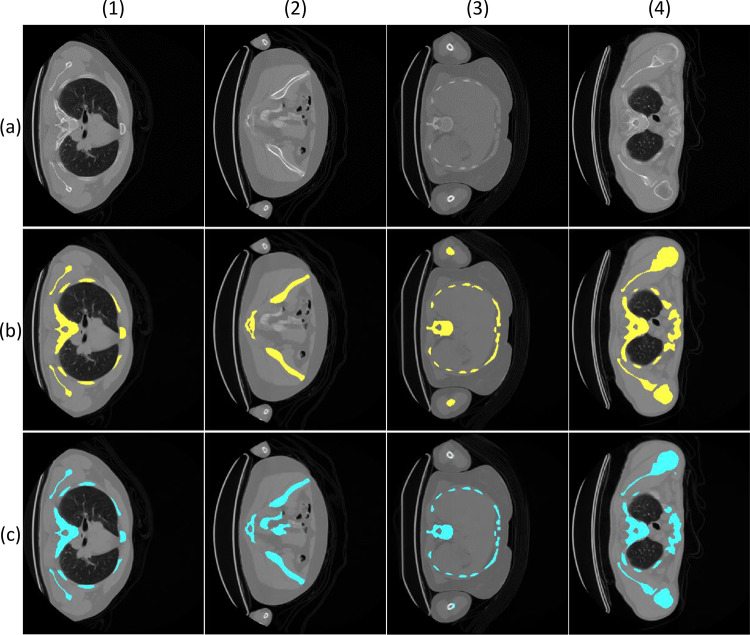
Experiment A segmentation results. The letters on the left edge indicate the type of image: **a** axial slice of the original CT, **b** ground truth overlaid on the slice of a, **c** segmentation resulting from Experiment A overlaid on the slice of a. The upper numbers indicate different cases: (1) correct segmentation; (2) segmentation with misleading structures: slice with organs with low attenuation in the CT, i.e., with high gray intensity levels; (3) segmentation with missing structures: slice with poorly segmented humeri; (4) segmentation with incomplete structures: slice with areas of cortical bone of small thickness together with bone marrow with gray intensity levels very similar to soft tissues

The best performance is obtained for Experiment A for the set group *F* = 8, *E* = 10, and the tenth order, with IoU (mask) = 0.920, IoU = 0.959, Dice (mask) = 0.958, and Dice = 0.979. As a result, this model is used to obtain and assess the segmentation results, shown in Fig. 8. As it can be seen, the general segmentation (Fig. 8c) obtains similar results compared to the ground truth (Fig. 8b). Even for the segmentation examples with misleading or missing structures obtained from the model and compared to the ground truth (Fig. 8c, columns 2, 3, and 4), most of the skeleton area is correctly segmented.

## Discussion

The segmentation of the skeleton can be very useful for multiple clinical applications such as the evaluation of bone metastases, the assessment of oncological therapies, or the measurement of bone dosimetry. In this study, a 2D skeleton segmentation model from axial CT slices is proposed.

The robustness of the proposed methodology, including both model building based on the U-Net architecture described in Ronneberger et al. [9] and preprocessing, is demonstrated by the similarity of the performance of the models and by the improvement in the performance compared to models obtained with the unprocessed CT scans with a Dice index of 0.564 (see Supplementary Material. A). As it can be seen in Fig. 5, the maximum performance values obtained for each pair of parameters *F* and *E*, varying the order of entry of the sets during training, are very similar. On the one hand, the study of the variation of the *F* parameter shows that the greater the number of channels of the initial feature map, and consecutively the rest of the neurons that make up the network, the higher the performance, i.e., the more complex the model, the better the performance. On the other hand, it can be observed that for all values of *F*, the results obtained with 5 epochs are lower than those obtained with 10 and 20 epochs, the latter being practically the same. For this reason, and with the aim of avoiding an overfitting in the model, a test with a higher number of this parameter is considered unnecessary for this study.

The similarity of the results between Experiment A and Experiment B is remarkable. As in Fig. 5, Fig. 7 shows the same trend for the parameter representing the number of epochs (*E*). However, for the parameter *F*, a very similar performance is obtained for values 4 and 8. Nonetheless, the performance of the models of Experiment A is slightly superior to those of Experiment B, demonstrating that intraset normalization generates a more representative training dataset than intrapatient normalization. In addition, the possibility of obtaining convergent models is greater in the case of normalization between a larger number of slices than in a normalization only between the slices from the same patients.

Finally, the best-performing model is the one obtained in Experiment A for *F* = 8 and *E* = 10, in line with the trends observed in the individual study of each parameter. The performance of this model achieves an IoU = 0.959 and a Dice = 0.979. The performance surpasses the segmentation algorithm developed in Milara et al. [12] (which obtains values of IoU of 0.811 and Dice of 0.947, calculated with the group of test used in the present study and the same ground truth) and the study of Pérez-Carrasco et al. [7]. The reason for this improvement is due to the fact that both studies apply image processing based solely on morphological processing based on gray intensity level, whereas the models developed in the present study are based on more complex features.

In terms of the application of deep learning for skeleton segmentation, the present study exceeds the results obtained by Klein et al. [8]. Although the study by Klein et al. uses a very similar network architecture, it develops a more complex model, i.e., of higher *F*. As seen above, the performance of the model should be higher when using a higher *F* value, but it achieves lower performance. The difference in performance may be due to (1) the lack of robustness of the dataset compared to the present study (77 CT volumes randomly mixed in terms of slices and patients vs. 18 in the study of Klein) and (2) the application of preprocessing for stretcher removal. On the other hand, the study by Noguchi et al. [10] uses a network architecture also based on U-Net less complex than the study by Klein et al., but more than the present one. The resulting model shows better performance than the model of the present study, possibly due to the robust data augmentation methodology implemented.

For the model implemented and defined as the best performing model, the following limitations can be observed in Fig. 8. Firstly, segmentation of misleading structures (Fig. 8 case 2). In cases where there are structures with a high level of gray intensity such as internal organs in rare cases and medical devices (catheters, prostheses, etc.), the model segments these structures as part of the skeleton segmentation. Secondly, segmentation with missing structures (Fig. 8 case 3). Especially for the humerus area, the ground truth segmentation includes the humerus mask only from the elbow and when it appears completely, i.e., when it is not covered or partially covered by the scanner structure, but in CT, the bone is present much earlier, even from the hand area. As a result, the model can overlook the humeri as part of the skeleton mask. And finally, segmentation with incomplete structures (Fig. 8 case 4). For the case of bones that have a cortical region of very limited thickness and a bone marrow region with very low levels of gray intensity, the model can overlook some low-gray-level regions, obtaining an incomplete segmentation of a structure.

In addition to the limitations of the model, it is worth mentioning the limitations of the methodology implemented. Firstly, due to limited computational resources (GPU-VRAM 6 GB), it is not possible to develop a U-Net network architecture with a higher value of *F*. Secondly, although the automatic preprocessing and segmentation of a CT volume take approximately 32 s, the training of all models (Experiments A and B, with three different values of *F*, with three different values of the number of epochs, and with ten training orders) has taken a total of 279 h, which is a large computational time in the development of the study. Thirdly, the limitation of the model is given not only by the computational or temporal resources but also by the architecture used since, in the present study, the U-Net network is tested for segmentation, while many other architectures could be used for the same purpose: DeepLab [14], Fully Convolutional Network [15], or variants of the U-Net itself [16].

Finally, dataset limitations must be mentioned. Despite having a total of 25,117 samples as it is a two-dimensional segmentation, the samples come from only 77 volumetric images, all acquired with the same scan. Furthermore, considering that 15% (12 patients with 3813 samples) of the total dataset have been dedicated to the test, the number of samples where the model is evaluated is also a significant limitation of the study. Additionally, the lack of data augmentation processes due to the considerable robustness of the present study dataset can be considered another limitation in the methodology.

Overall, the model implemented and proposed in the present study achieves a proper performance in the CT skeleton segmentation with an IoU of 0.959 and a Dice of 0.979. Future works lead to improving model performance through the reduction of randomization in the definition of the models (avoiding non-convergent models) using initial seed of randomization and data augmentation methodologies. In addition, the study aims to be assessed with CT scans of patients diagnosed with different pathologies which also affect the bone structure. When the best-performing segmentation model is obtained, quantification models of the bone structures to observe their loss of volume or appearance of lesions will be proposed. Consequently, the final aim of the proposed methodologies and the proposed future works is using the developed models in a clinical tool as a clinical support decision system for the segmentation and quantification of the skeleton in multiple clinical contexts which affect the bone structures.

## Conclusions

The skeleton segmentation can be useful for multiple clinical purposes. In this study, a neural network model based on the U-Net architecture for the skeleton segmentation from the CT slices is proposed. Different parameters are varied and tested to obtain the best possible model. With high performance, it demonstrates the potential of deep learning applied in medical images.

## Supplementary Information

Below is the link to the electronic supplementary material.

Supplementary file1 (DOCX 1796 KB)
