# Supplementary material for: Automatic Skeleton Segmentation in CT Images Based on U-Net
Source: J Imaging Inform Med. 2024 Apr 30;37(5):2390–400. doi: 10.1007/s10278-024-01127-5 (PMC11522221; doi:10.1007/s10278-024-01127-5)
Supplement: Supplementary file 1 — Supplementary file1 (DOCX 1796 KB) [file 10278_2024_1127_MOESM1_ESM.docx]

**Automatic skeleton segmentation in CT images based on U-Net – Supplementary Material**

# Dice(mean) index results for no-processed CT scans

Previous to design the preprocessing steps, the models are performed with any preprocess, only clipping from the first femur slice to the last slice without head and the datasets randomization. These models result on an unacceptable segmentation, shown in Fig. 1. The best performance obtained is a Dice index of 0.564.


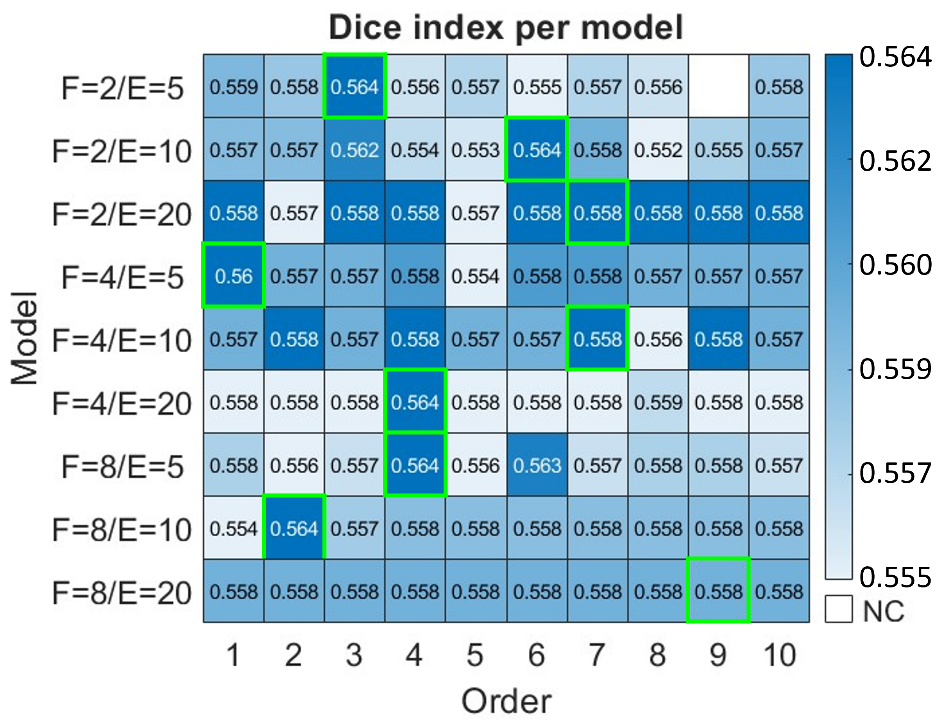


**Fig. 1.** Comparison of training orders for each model, i.e., different pairs of F and E in no-processed database. NC: Non-Convergent. In lighting green are shown the best result for each pair of feature models: the best Dice Index; a same value of Dice Index, the best Dice (mask) Index; a same value of Dice (mask) Index, the model with higher value of TP.

Once the best performance model for non-processed CT scan is selected, the segmentation problems are observed in order to design the preprocessing steps. The main segmentation mistakes are shown in Fig. 2. Due to the grey intensity levels, only pixels with high grey intensity levels are included in the segmentation, causing not only an exclusion of regions with low grey intensity levels such as bone marrow (Fig. 2a) or bone with reduced thickness (Fig. 2b), but also an over-segmentation of organs with high Hounsfield Units (Fig. 2c) or regions such as stretcher (Fig. 2d).

| 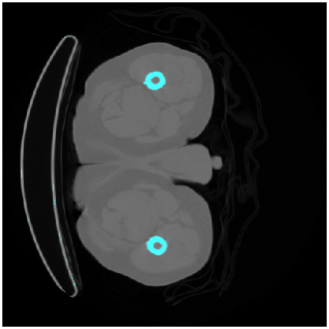(a) | 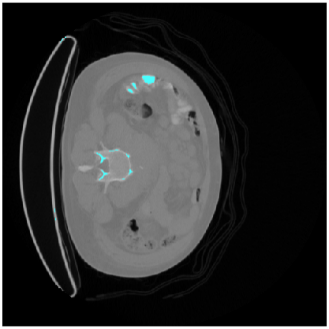(b) | 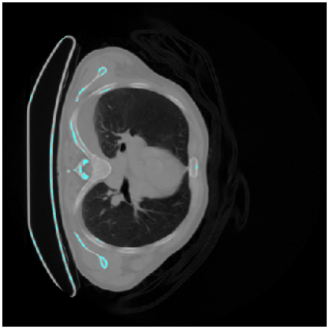(c) | 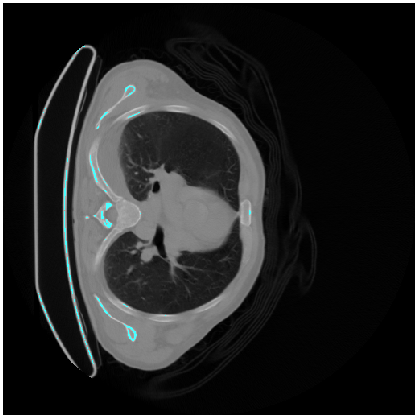(d) |
| --- | --- | --- | --- |

**Fig. 2.** Skeleton segmentation mistakes for no-processed database. (a) Lack of bone marrow in skeleton segmentation. (b) Organs with higher grey intensity levels than bone inclusion. (c) Exclusion of cortical bone regions of low grey intensity levels i.e. reduced thickness. (d) Stretcher inclusion due to its high grey intensity level.

# Complete stretcher removal process

Fig. 3, Fig. 4 and Fig. 5abc show the step-by-step process of stretcher removal. First, the CT image (Fig. 3a) is binarized, converting all values greater than 98 HU to 1 and the rest to 0 (Fig. 3b).

From the binarized image, the point closest to the image margin in the stretcher direction, i.e., at the left edge, is located along with the vertical line which cross this point (i0_1, shown in red in Fig. 3c). Assuming this vertical line as the beginning of the stretcher, the next margin of the stretcher in the horizontal central zone that is included in the binary image, thus eliminating the soft zone of the stretcher, is located along with its vertical line (i0_2, shown in yellow in Fig. 3c). Finally, to include also the extremes of the stretcher, a third index is obtained, which is the result of adding 20 pixels to the central index, and again the vertical line which cross this point is obtained (i0, shown in green in Fig. 3c). Based on the calculated indexes, it is estimated whether the whole stretcher is shown (difference between i0_1 and i0_2 over 50 pixels) or only the upper half of it (otherwise) in order to calculate the indexes that define the ends of the stretcher in the central zone, in the curved zone and in the limits of the stretcher (i1, i2 and i3, shown as its vertical lines in green, yellow and red, respectively, in Fig. 3d). In case the complete stretcher is contained in the image, the indexes are calculated following (1). Otherwise, following (2).

$Complete stretcher\left\{ \begin{aligned} i1=105+i0 \\ i2=i1-20 \\ i3=i2-10 \end{aligned} \right.$ (1) $Half of stretcher\left\{ \begin{aligned} i1=45+i0 \\ i2=i1-10 \\ i3=i2-10 \end{aligned} \right.$ (2)

Subsequently, the axial slice is divided in its vertical direction into 8 equal regions R1-R8, from top to bottom (shown in blue in Fig. 3e) divided by its correspondent horizontal lines. The first 2 regions (R1 and R2) and the last two (R7 and R8) are associated with index i3, i.e., the ends of the stretcher. In this way, the intersection points between the horizontal lines that limit R2 below and R7 above with the vertical line corresponding to the index i3 are found (Pe1 and Pe2, respectively, shown in white in Fig. 3f). The central regions (R4 and R5) are associated with index i1, i.e., the central zone. In the same way, the intersection points of the horizontal lines that limit R4 superiorly and R5 inferiorly with the vertical line corresponding to index i1 are also found (Pe3 and Pe4, respectively, shown in white in Fig. 3f). Finally, the regions with the highest inclination, the transit regions between the central and the end regions (R3 and R6) are associated with index i2. In order to ensure that the margin is formed by vertical and horizontal lines and that no tilt confuses stretcher pixels on the other side, in this case the points of intersection of all the horizontal lines named in the previous cases (below R2, above R7, above R4 and below R5) with the vertical line corresponding to the index i2 are found (Pi1, Pi2, Pi3 and Pi4, respectively, shown in pink in Fig. 3f). Then, to create the margin separating the stretcher from the body of the patient, the intersection points defined are joint as follows: the upper point of the line defined by the index i3 (at the upper margin of the image) with Pe1; from this point join the intersection points in the following order Pe1 - Pi1 - Pi3 - Pe3 - Pe4 - Pi4 - Pi2 - Pe2; and finally Pe2 with the lower point defined by the line i3 (at the lower margin of the image) as shown in (Fig. 3g). In this way, the margin separating the stretcher from the skeletal area is obtained (Fig. 3h).

| 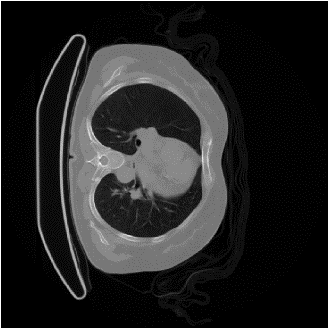(a) | 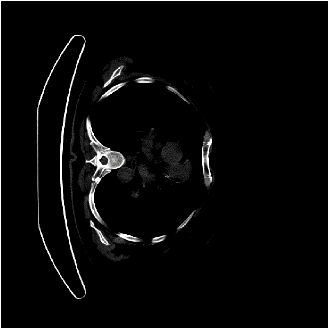(b) | 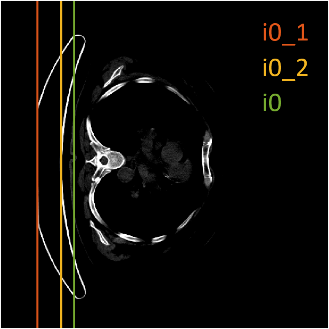(c) | 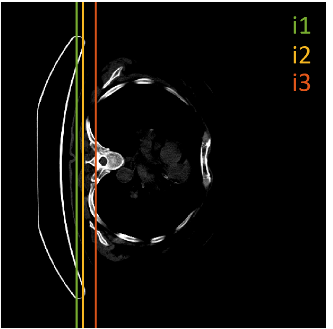(d) |
| --- | --- | --- | --- |
| 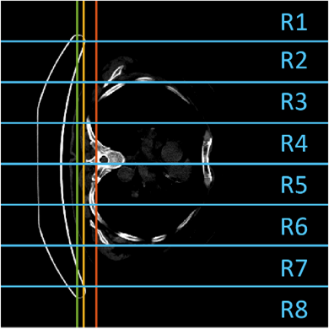(e) | 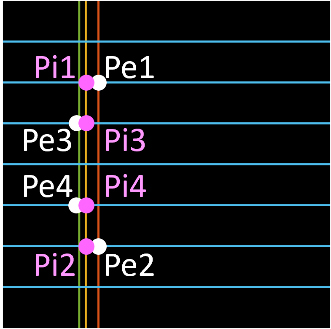(f) | 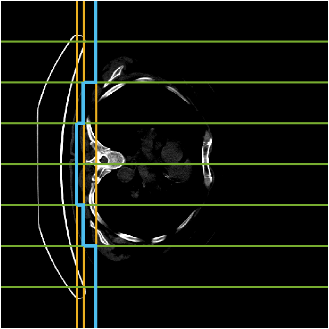(g) | 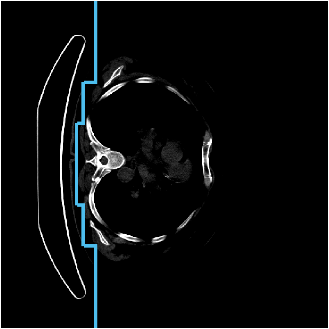(h) |

**Fig. 3.** Stretcher removal I: margin location. (a) Axial slice of the original CT scan of the torso area. (b) Slice threshold between 0 and 300 gray levels. (c) Location of the stretcher points over the CT slice: closest to the left image margin in the horizontal central zone (i0_1, red), furthest from the left image margin in the central zone (i0_2, yellow), the point representing where most of the elevation of the stretcher ends (i0, green). (d) Location of the vertical lines that separate the stretcher from the body of the patient: in the central zone (i1, green), in the curve that goes from the central zone to the ends of the stretcher (i2, yellow), and at these ends (i3, red). (e) Horizontal division of the slice in 8 regions of identical size (R1-R8, blue). (f) Intersection points from Pe1 to Pe4 and Pi1 to Pi4 shown over the correspondent vertical and horizontal lines (CT scan is not included for getting an easier visualization). (g) Generation of the margin (blue) of separation between body and stretcher based on the previously calculated regions (green) and indices (yellow). (h) Margin (blue) separating the stretcher from the skeletal area shown in the CT slice.

Once the margin separating stretcher from the skeletal regions is obtained, the binarized image is searched for pixels in the region between the left border and the margin calculated and defined as the mask corresponding to the stretcher. In addition, especially for the cases where the middle of the stretcher appears, the first 20 rows of pixels, i.e., the closest pixels to the left border, are also defined as the stretcher (Fig. 4a) to facilitate the filling process of the following step (b). Subsequently, not only the hole generated by the soft tissue of the stretcher, but also the space between the stretcher and the left margin, are filled for obtaining a single structure (Fig. 4b). Taking into account that not anatomical regions should be included below the stretcher, the space between superior and inferior image margins and the stretcher is filled too (Fig. 4c). Finally, two morphological operations are performed to avoid bigger holes and to avoid missing the borders of the structure (Fig. 4d).

| 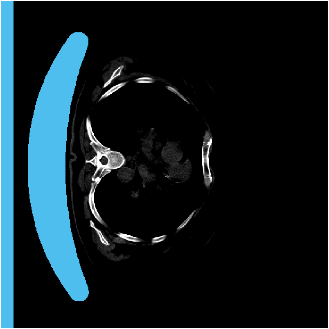(a) | 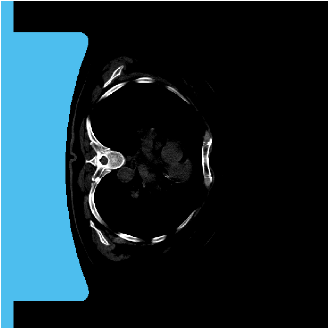(b) | 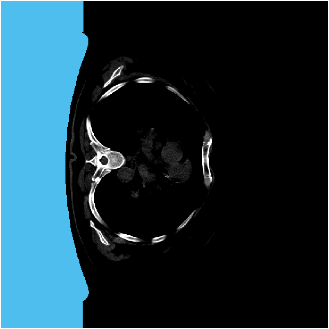(c) | 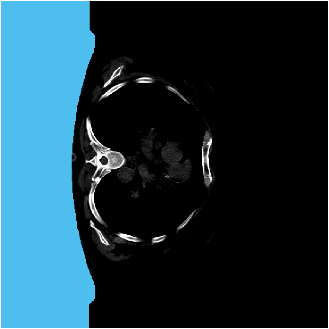(d) |
| --- | --- | --- | --- |

**Fig. 4.** Stretcher removal II: stretcher filling. Blue mask represents the stretcher mask. (a) Threshold slice of the CT scan highlighting the pixels with intensity level higher or equal to 98 in the region defined by the left border and the calculated margin. (b) Stretcher mask after filling holes of the mask. (c) Stretcher mask after filling the area between stretcher and superior and inferior border. (d) Stretcher mask after filling holes of the mask.

Finally, this mask is adapted to every slice of the same patient. For this purpose, the pixels classified as stretch mask are assigned as 0 in the binarized image. Then, slice-by-slice, the dilated mask, with a structuring element of circular shape and radius 7, is checked to see if it coincides in any pixel with the initial binarized mask that contained both the stretcher and the skeleton. If so, the initial shape of the stretcher is maintained (Fig. 5a). Otherwise, the dilated shape of the stretcher is used (Fig. 5b, being the green region corresponding to dilatation). Finally, for the superior slices, the dilation is used to remove a stretcher accessory that is placed on some patients in the head region to prevent movement and improve their comfort. In this case, a slice in which the stretcher presents this accessory is found, and the lower and upper slices are searched to adjust the shape of the stretcher to the shape of the accessory (Fig. 5c). Once the stretcher mask is obtained, it is removed from the CT scan defining every pixel present in the stretcher mask as a 0 in the CT scan (Fig. 5d).

| 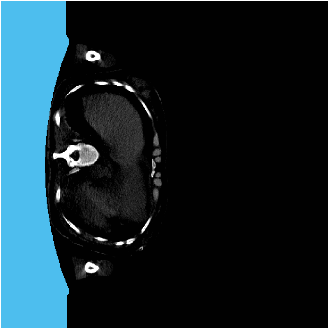(a) | 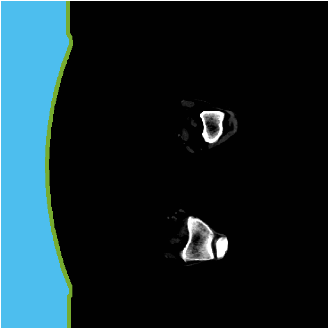(b) | 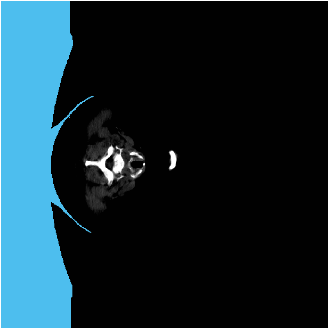(c) | 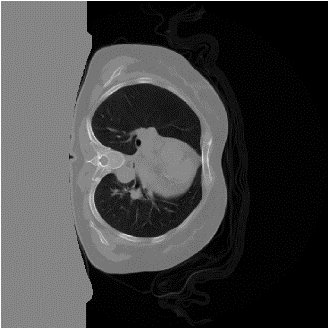(d) |
| --- | --- | --- | --- |

**Fig. 5.** Stretcher removal III: stretcher adaptation. The blue mask represents the stretcher mask. (a) Stretcher mask for a CT slice without adaptation. (b) Stretcher mask for a CT slice with adaptation by means of dilatation. The green mask represents the adaptation with respect to the original stretcher. (c) Stretcher mask for a CT slice with adaptation by means of stretcher accessory searching. (d) Original CT scan slice substituting the values of the stretcher region by 0.

# Kruskal-Wallis and Post hoc analysis for groups

To confirm that the models obtained show statistically significant differences in the bone segmentation, a Kruskal-Wallis analysis is performed. For this purpose, the Dice (mean) is calculated for each of the slices used in the test for each pair of F/E values in the order of highest performance, obtaining a total of 3813 values for each model. Then, Kruskal-Wallis *p*-values are calculated for each experiment, being both <0.001. Boxplot of this index results and the respective Kruskal-Wallis *p*-value are included in Fig. 6 and Fig. 7.


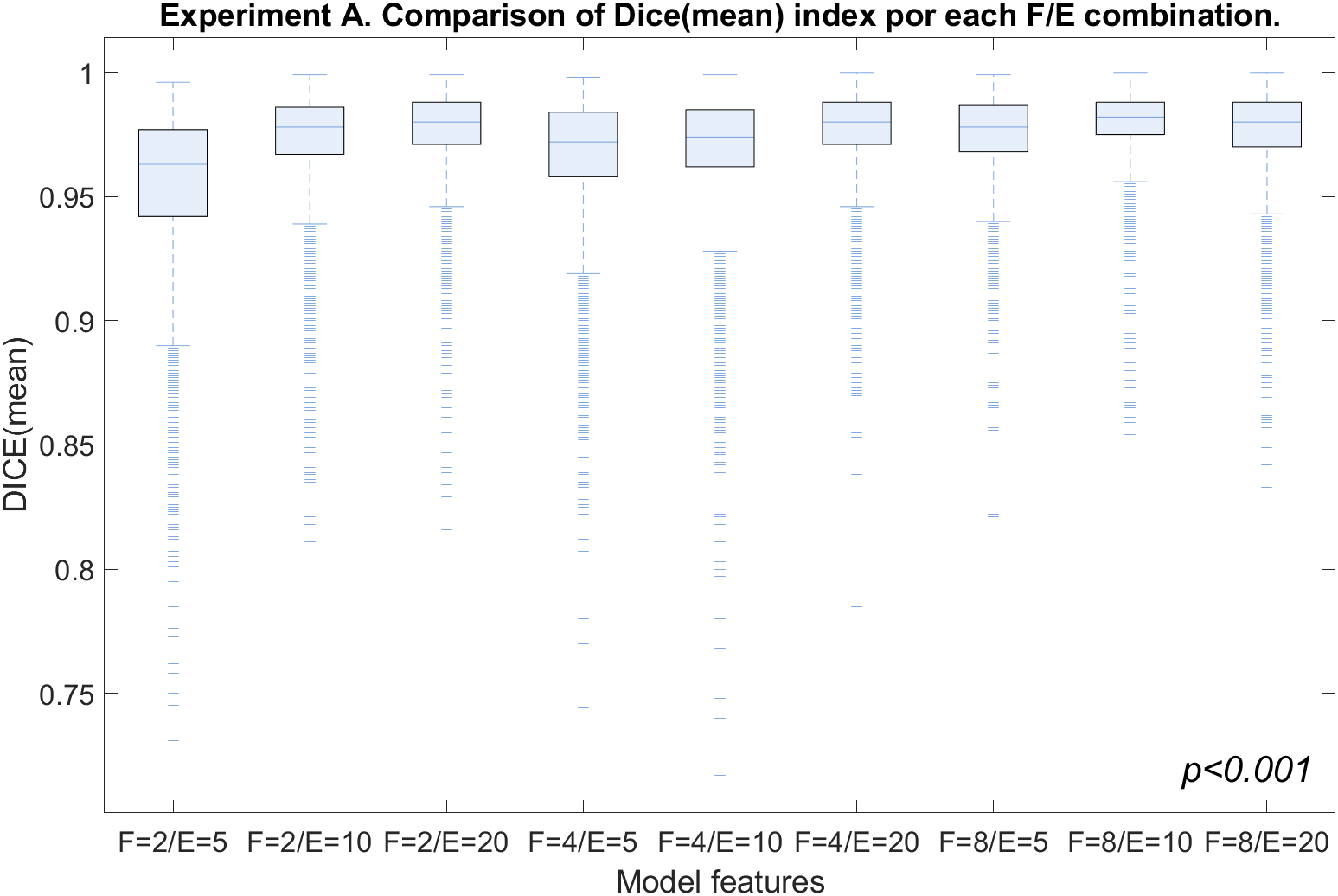


**Fig. 6.** Experiment A. Boxplot of Dice(mean) index for each pair of F/E features. Kruskal-Wallis p-value is included in the bottom-right corner of the plot.


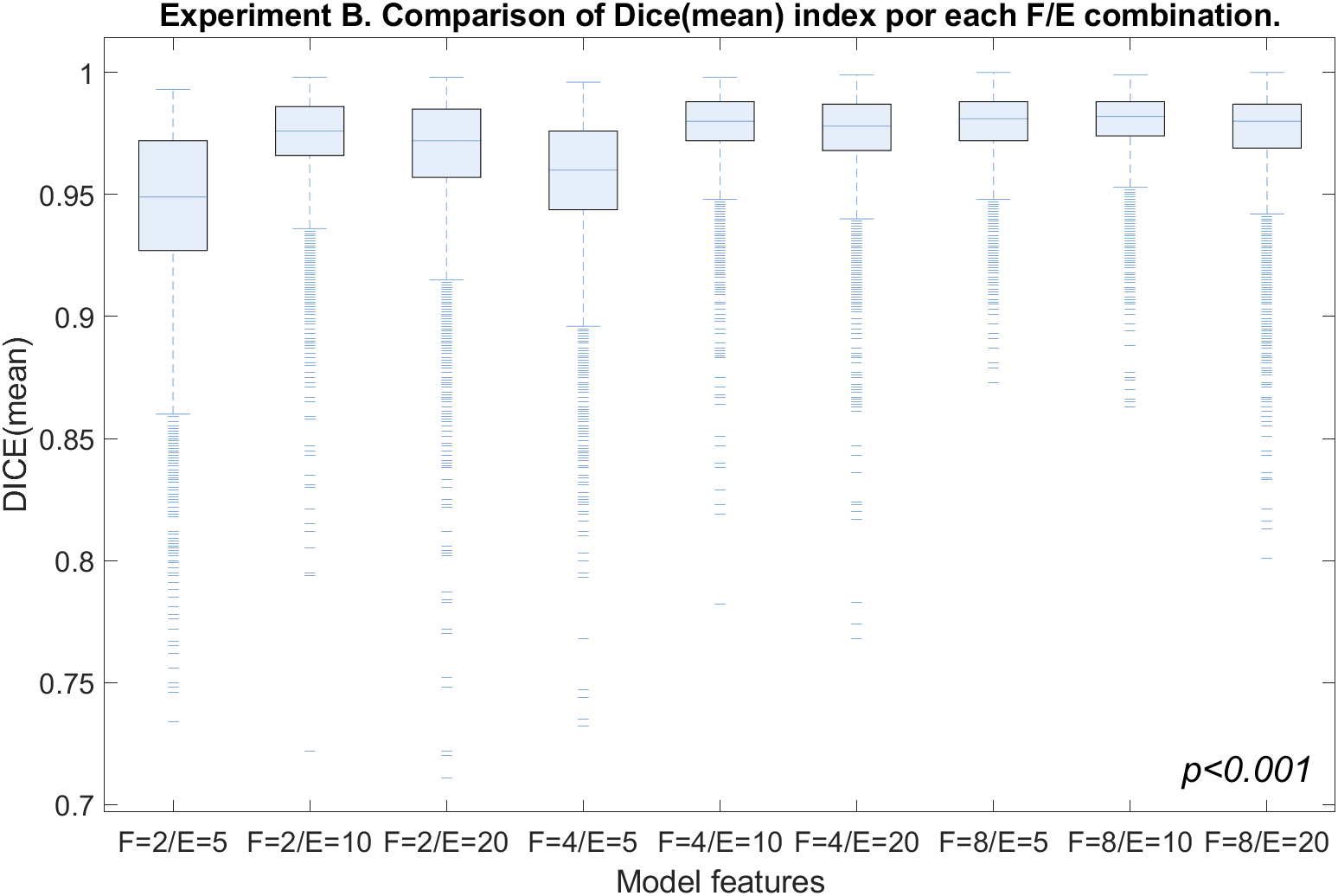


**Fig. 7.** Experiment B. Boxplot of Dice(mean) index for each pair of F/E features. Kruskal-Wallis p-value is included in the bottom-right corner of the plot.

In addition, to confirm that statistically significant differences are also found each feature value, the Kruskal-Wallis analysis is performed for the different values of F (2, 4 and 8) by varying E, and in the same way with the parameter E by varying F. Both tests are performed for the two experiments. As it can be seen in Fig. 8 and Fig. 9, for Experiment A, all tests present a *p*-value<0.001, except from the experiment for E=20, where a statistically significant difference cannot be proved.


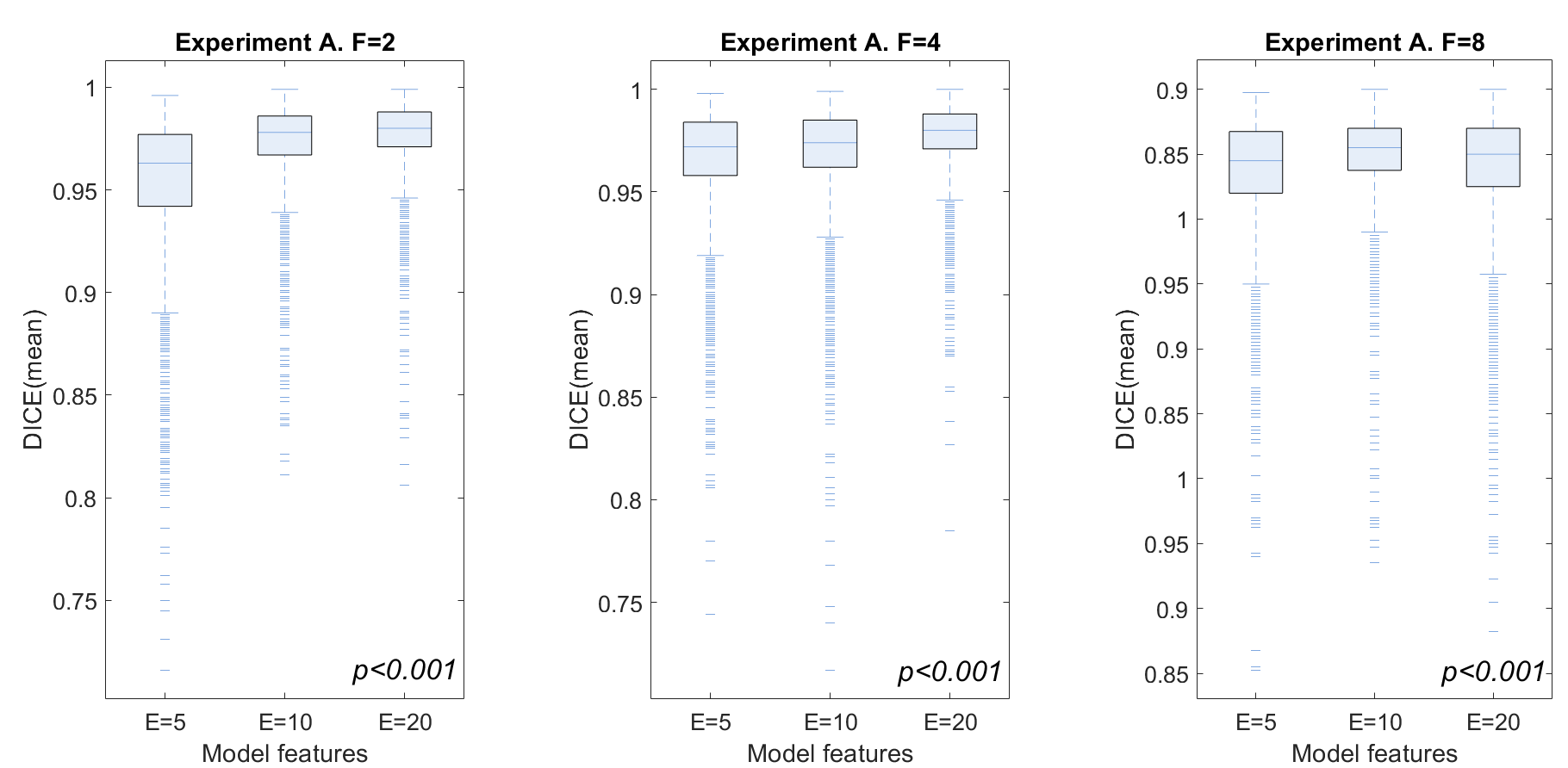


**Fig. 8.** Experiment A. Boxplot of Dice(mean) index for each F value varying E. Kruskal-Wallis p-value is included in the bottom-right corner of the plot.


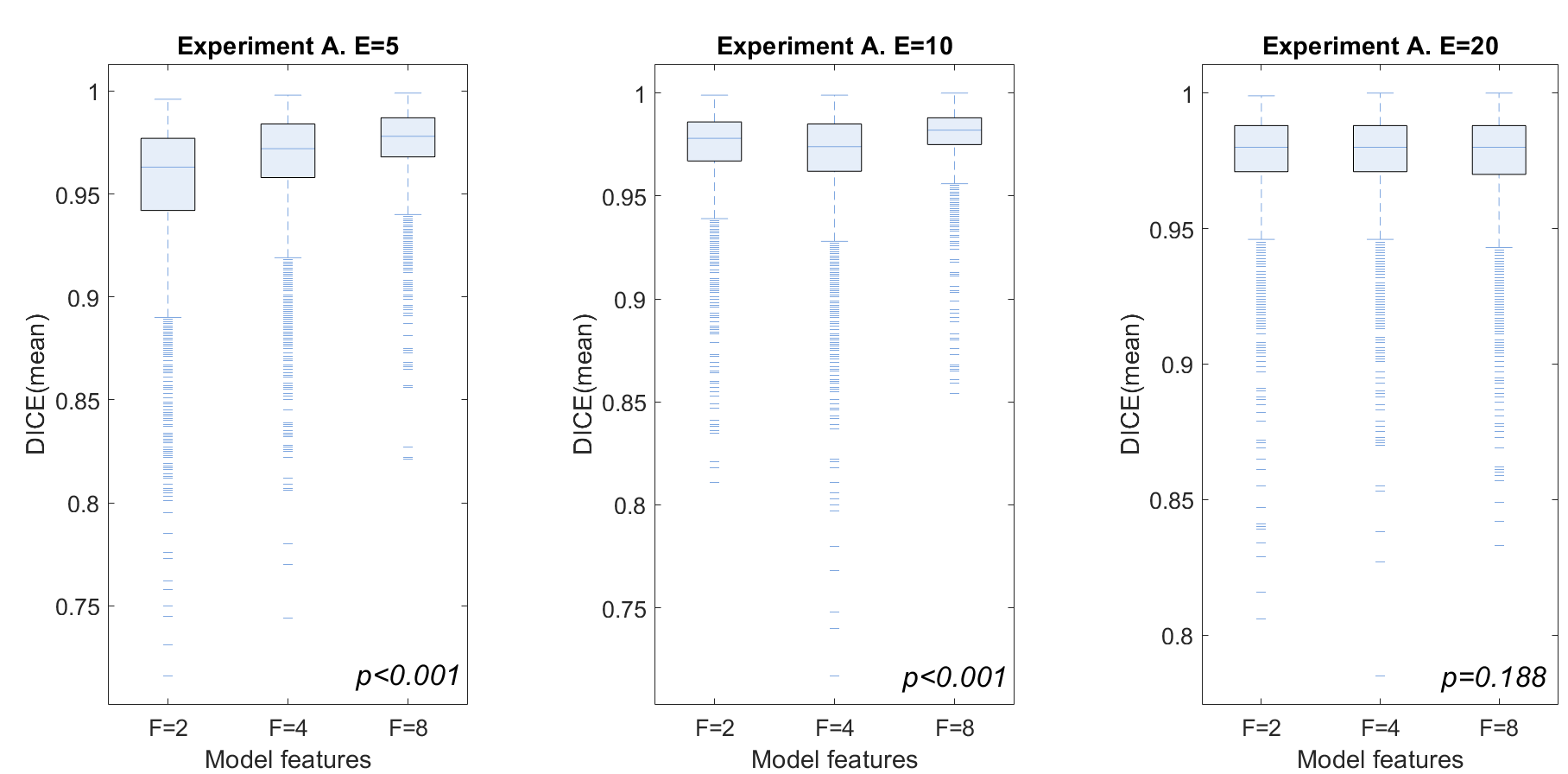


**Fig. 9.** Experiment A. Boxplot of Dice(mean) index for each E value varying F. Kruskal-Wallis p-value is included in the bottom-right corner of the plot.

On the other hand, Experiment B shows statistically significant differences for any value of model features (Fig. 10 and Fig. 11).


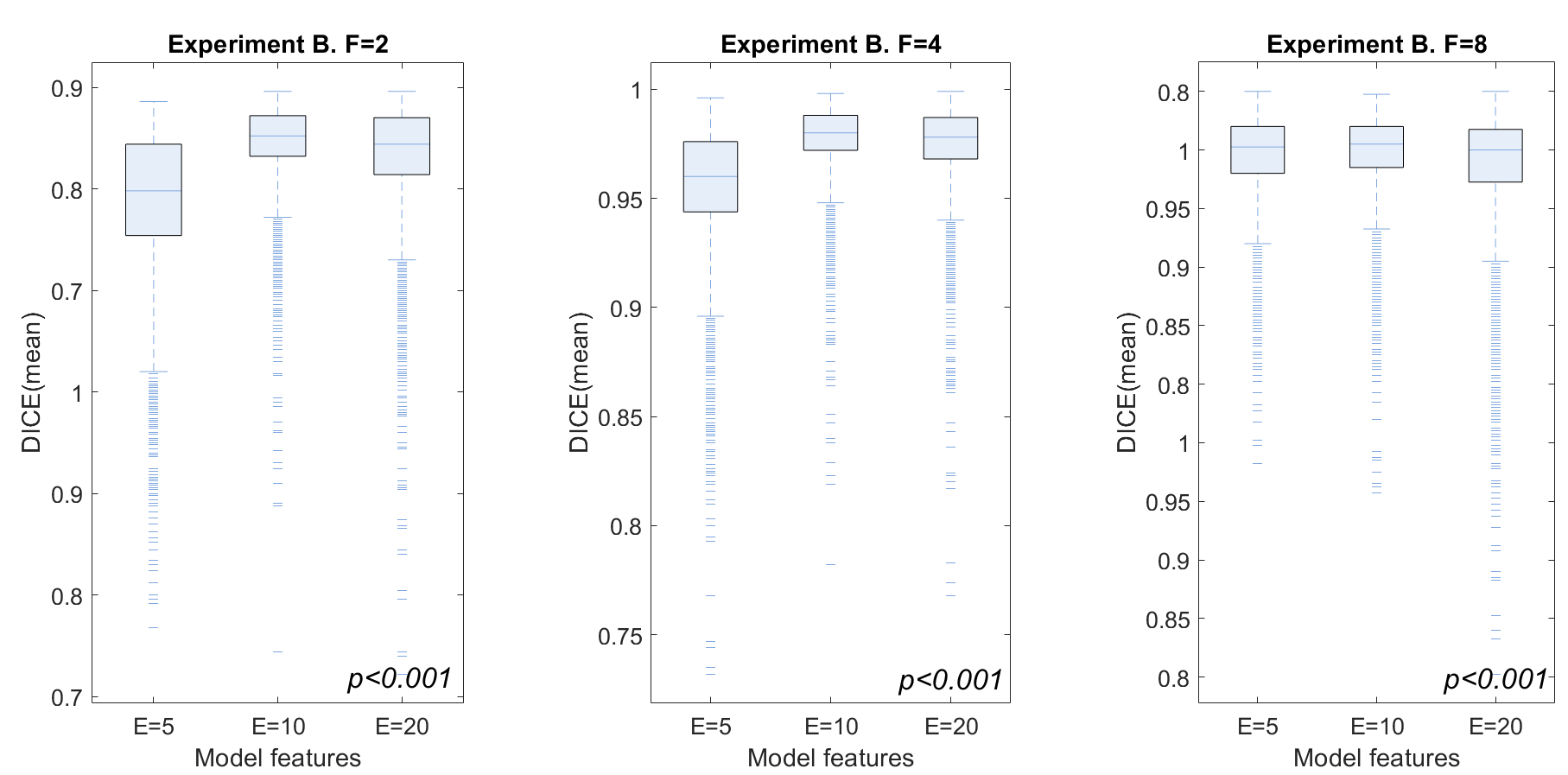


**Fig. 10.** Experiment B. Boxplot of Dice(mean) index for each F value varying E. Kruskal-Wallis p-value is included in the bottom-right corner of the plot.


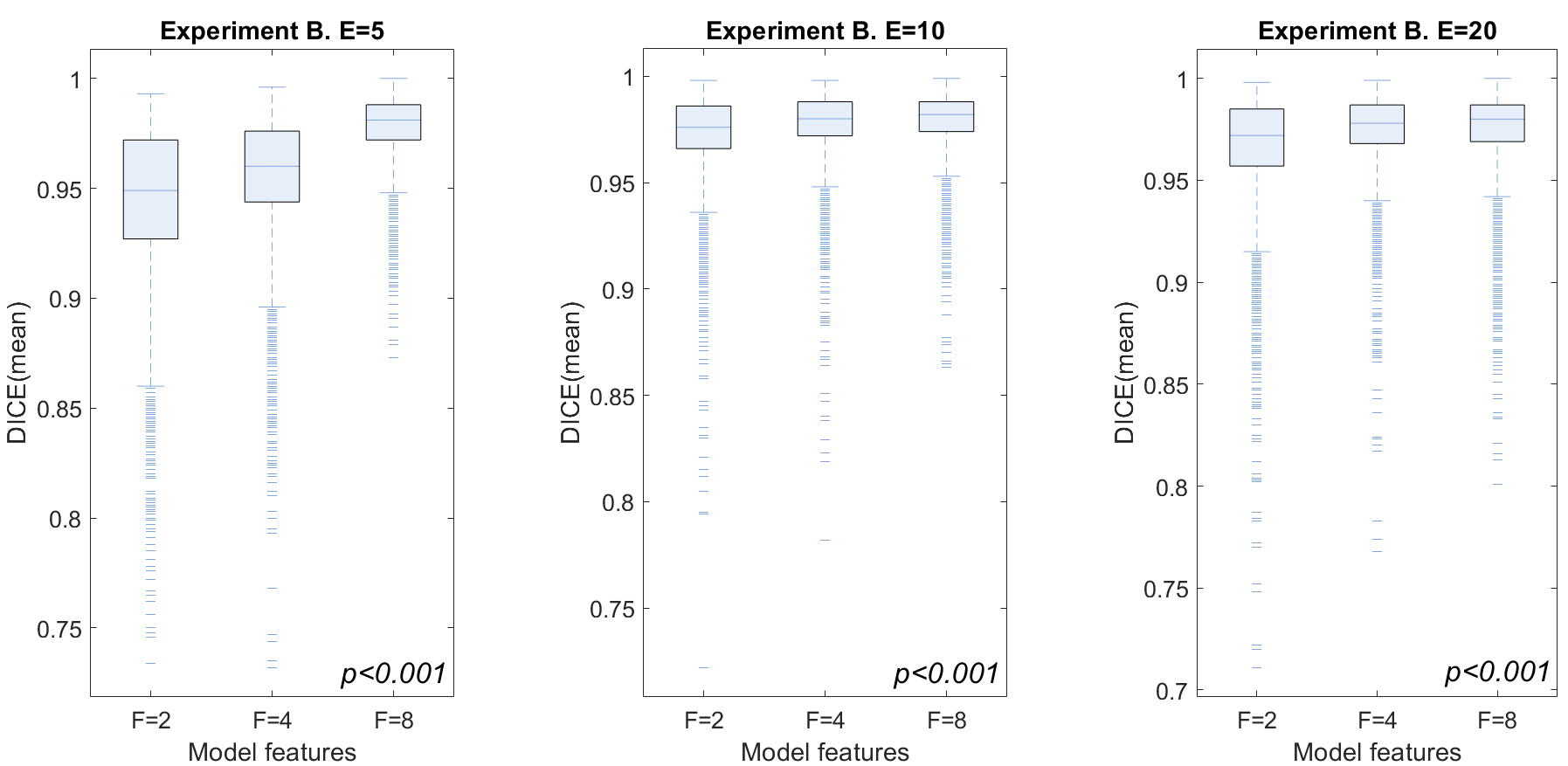


**Fig. 11.** Experiment B. Boxplot of Dice(mean) index for each E value varying F. Kruskal-Wallis p-value is included in the bottom-right corner of the plot.

Taking into consideration that the vast majority of groups present statistically significant differences, post hoc analyses are also performed and corrected by means of Benjamini-Hochberg procedure. For Experiment A, as it can be seen in Table 1 and following with the Kruskal-Wallis results, the lack of statistically significant differences between models characterized by E = 20 is presented between each of the models (different F). Furthermore, post hoc tests are performed for Experiment A for each F value and E value, similarly to Kruskal-Wallis analysis. However, all *p*-values are <0.05.

| Pair of F/E | F=2/ E=5 | F=2/ E=10 | F=2/ E=20 | F=4/ E=5 | F=4/ E=10 | F=4/ E=20 | F=8/ E=5 | F=8/ E=10 | F=8/ E=20 |
| --- | --- | --- | --- | --- | --- | --- | --- | --- | --- |
| F=2/E=5 | X | <0.001 | <0.001 | <0.001 | <0.001 | <0.001 | <0.001 | <0.001 | <0.001 |
| F=2/E=10 | <0.001 | X | <0.001 | <0.001 | <0.001 | <0.001 | 0.041 | <0.001 | <0.001 |
| F=2/E=20 | <0.001 | <0.001 | X | <0.001 | <0.001 | **1** | <0.001 | <0.001 | **1** |
| F=4/E=5 | <0.001 | <0.001 | <0.001 | X | <0.001 | <0.001 | <0.001 | <0.001 | <0.001 |
| F=4/E=10 | <0.001 | <0.001 | <0.001 | <0.001 | X | <0.001 | <0.001 | <0.001 | <0.001 |
| F=4/E=20 | <0.001 | <0.001 | **1** | <0.001 | <0.001 | X | <0.001 | <0.001 | **1** |
| F=8/E=5 | <0.001 | 0.041 | <0.001 | <0.001 | <0.001 | <0.001 | X | <0.001 | <0.001 |
| F=8/E=10 | <0.001 | <0.001 | <0.001 | <0.001 | <0.001 | <0.001 | <0.001 | X | <0.001 |
| F=8/E=20 | <0.001 | <0.001 | **1** | <0.001 | <0.001 | **1** | <0.001 | <0.001 | X |

**Table 1.** Experiment A. Post hoc *p-*values for each pair of groups. Bold is used for *p*-values>0.05.

On the other hand, for Experiment B, Table 2 complete the information obtained with Kruskal-Wallis test. For all models, statistically significant differences are observed, except from 3 pair of models. In the case of the pair F=8/E=10 and F=8/E=5, the *p*-value obtained (>0.05) represent the difference of F=8 group of models in the model defined by F=8/E=20. Similarly, the pair F=8/E=20 and F=4/E=20, represent the difference of F=2/E=20 model in the E=20 models group.

| Pair of F/E | F=2/  E=5 | F=2/  E=10 | F=2/  E=20 | F=4/  E=5 | F=4/  E=10 | F=4/  E=20 | F=8/  E=5 | F=8/  E=10 | F=8/  E=20 |
| --- | --- | --- | --- | --- | --- | --- | --- | --- | --- |
| F=2/E=5 | X | <0.001 | <0.001 | <0.001 | <0.001 | <0.001 | <0.001 | <0.001 | <0.001 |
| F=2/E=10 | <0.001 | X | <0.001 | <0.001 | <0.001 | 0.003 | <0.001 | <0.001 | <0.001 |
| F=2/E=20 | <0.001 | <0.001 | X | <0.001 | <0.001 | <0.001 | <0.001 | <0.001 | <0.001 |
| F=4/E=5 | <0.001 | <0.001 | <0.001 | X | <0.001 | <0.001 | <0.001 | <0.001 | <0.001 |
| F=4/E=10 | <0.001 | <0.001 | <0.001 | <0.001 | X | <0.001 | **0.82** | 0.002 | 0.004 |
| F=4/E=20 | <0.001 | 0.003 | <0.001 | <0.001 | <0.001 | X | <0.001 | <0.001 | **0.44** |
| F=8/E=5 | <0.001 | <0.001 | <0.001 | <0.001 | **0.82** | <0.001 | X | **1** | <0.001 |
| F=8/E=10 | <0.001 | <0.001 | <0.001 | <0.001 | 0.002 | <0.001 | **1** | X | <0.001 |
| F=8/E=20 | <0.001 | <0.001 | <0.001 | <0.001 | 0.004 | **0.44** | <0.001 | <0.001 | X |

**Table 2.** Experiment A. Post hoc *p-*values for each pair of groups.
